# Random survival forests for dynamic predictions of a time-to-event outcome using a longitudinal biomarker

**DOI:** 10.1186/s12874-021-01375-x

**Published:** 2021-10-17

**Authors:** Kaci L Pickett, Krithika Suresh, Kristen R Campbell, Scott Davis, Elizabeth Juarez-Colunga

**Affiliations:** 1 Department of Pediatrics, University of Colorado Anschutz Medical Campus, Aurora, 80045 Colorado USA; 2 Department of Biostatistics and Informatics, University of Colorado Anschutz Medical Campus, Aurora, 80045 Colorado USA; 3 Adult and Child Consortium for Health Outcomes and Delivery Science, University of Colorado Anschutz Medical Campus, Aurora, 80045 Colorado USA; 4 Division of Renal Diseases and Hypertension, University of Colorado Anschutz Medical Campus, Aurora, 80045 Colorado USA

**Keywords:** Area under the curve, Joint modeling, Landmarking, Prediction accuracy, Variable importance

## Abstract

**Background:**

Risk prediction models for time-to-event outcomes play a vital role in personalized decision-making. A patient’s biomarker values, such as medical lab results, are often measured over time but traditional prediction models ignore their longitudinal nature, using only baseline information. Dynamic prediction incorporates longitudinal information to produce updated survival predictions during follow-up. Existing methods for dynamic prediction include joint modeling, which often suffers from computational complexity and poor performance under misspecification, and landmarking, which has a straightforward implementation but typically relies on a proportional hazards model. Random survival forests (RSF), a machine learning algorithm for time-to-event outcomes, can capture complex relationships between the predictors and survival without requiring prior specification and has been shown to have superior predictive performance.

**Methods:**

We propose an alternative approach for dynamic prediction using random survival forests in a landmarking framework. With a simulation study, we compared the predictive performance of our proposed method with Cox landmarking and joint modeling in situations where the proportional hazards assumption does not hold and the longitudinal marker(s) have a complex relationship with the survival outcome. We illustrated the use of the RSF landmark approach in two clinical applications to assess the performance of various RSF model building decisions and to demonstrate its use in obtaining dynamic predictions.

**Results:**

In simulation studies, RSF landmarking outperformed joint modeling and Cox landmarking when a complex relationship between the survival and longitudinal marker processes was present. It was also useful in application when there were several predictors for which the clinical relevance was unknown and multiple longitudinal biomarkers were present. Individualized dynamic predictions can be obtained from this method and the variable importance metric is useful for examining the changing predictive power of variables over time. In addition, RSF landmarking is easily implementable in standard software and using suggested specifications requires less computation time than joint modeling.

**Conclusions:**

RSF landmarking is a nonparametric, machine learning alternative to current methods for obtaining dynamic predictions when there are complex or unknown relationships present. It requires little upfront decision-making and has comparable predictive performance and has preferable computational speed.

**Supplementary Information:**

The online version contains supplementary material available at (10.1186/s12874-021-01375-x).

## Background

Risk prediction models assist physicians in making personalized clinical care decisions for their patients. In predicting a survival outcome, prognostic models are traditionally developed using variables collected on patients at a baseline time. When the variables are measured multiple times over a patient’s follow-up, such as medical lab results, many models ignore the longitudinal trajectory of these markers and utilize only baseline values. Thus, these models fail to take into account how changes in the marker over time may affect risk. Ideally, a patient’s survival prediction should be updated dynamically as the values of the longitudinal marker change. Dynamic prediction incorporates time-dependent marker information collected during a patient’s follow-up to produce updated, more accurate estimates of their survival probability.

Two commonly used statistical approaches for dynamic prediction are joint modeling and landmarking. Joint modeling involves specifying a model for the longitudinal marker process, a model for the survival outcome, and uses a function to link the two [1, 2]. Estimation of this model and computation of the conditional survival probabilities involve numerical integration and can require substantial computation time [2, 3]. It also requires assumptions about the relationship between the marker process and event time that may be unknown, or even when known, often lead to convergence issues and pose a computational burden as the complexity increases [2]. When there is interest in incorporating multiple longitudinal markers, approximation techniques must be used to evaluate parameter estimates but extensions are limited as is the software to perform the computations [4, 5].

Landmarking requires specifying a sequence of survival models for the subsample of individuals still at risk at prediction times of interest during follow-up, referred to as landmark times. At each landmark time, the model incorporates the value of the longitudinal marker at that time, or with extension, the longitudinal marker history up to that time [6, 7]. In classic landmarking, a Cox proportional hazards model is used. This approach avoids specifying the distribution of the stochastic marker process in time, making it appealing compared to the distributional assumptions required by joint modeling. It has been shown that landmarking prediction accuracy is affected by misspecification of the dependence structure between the longitudinal process and the survival process, but is less sensitive to misspecification of the longitudinal marker trajectory and violation of the proportional hazards assumption [7–9].

Both joint modeling and landmarking have limitations when used to capture complex relationships between the marker and survival. These methods have yet to be explored for dynamic prediction in scenarios with a multitude of available patient and clinical information, where the quantity and complex relationships between these variables can present challenges for these classic techniques. If many variables are plausible candidates for affecting the survival outcome, and these relationships are largely unknown or complex, correctly specifying a model to describe their relationship with the survival outcome can be difficult. This complexity increases with multiple longitudinally-measured variables, for which the longitudinal marker trajectories, their dependence with the survival process, and their dependence with each other must be specified. Both joint modeling and landmarking also rely on a survival model with a proportional hazards assumption [6, 8], which can be restrictive and unrealistic in practice. Thus, we hypothesize that a nonparametric approach for dynamic prediction that does not require explicit specification of the covariate relationships can improve upon prediction accuracy and ease-of-implementation.

Machine learning algorithms are nonparametric methods that assume no prior knowledge about the data and have become popular in the prediction of survival outcomes [10–14]. These methods have been shown to have superior predictive performance compared to traditional regression methods when there are a large number of predictors relative to the sample size (high-dimensional data) and predictors have nonlinear, complex relationships in the hazard for the survival outcome [15, 16]. Dynamic prediction with machine learning has been explored with the use of a machine learning ensemble for binary outcomes adapted to survival data [17]. The predictive performance of this approach was similar to joint modeling and Cox landmarking in a data application. However, the method is complex and does not provide an interpretation of the covariate effects on survival. We propose a machine learning approach for dynamic prediction that uses the tree-based method of random survival forests and is easily implementable in standard software. This approach will provide an alternative way of obtaining accurate dynamic predictions in the presence of multiple covariates without need for prior specification of covariate relationships.

A random survival forest (RSF) is a nonparametric ensemble method for the analysis of right censored survival data, built as a time-to-event extension of random forests for classification [12, 18]. The method can handle multiple covariates, noise covariates, as well as complex, nonlinear relationships between covariates without need for prior specification [19]. As such, RSFs often fill a role in replacing classic Cox regression when the proportional hazards assumption is in question as it is assumption-free [20]. There is recent work that explores the use of RSF in the context of dynamic prediction. One such work includes an investigation of using a landmark-type model to predict a time-to-event based on a discrete-time survival model [21]. Another approach uses counting processes to dynamically predict risk based on a piece-wise constant hazard model [22]. A new definition of the receiver operating characteristic curve has also been developed for evaluating the performance of RSF [23]. Approaches to handle multiple longitudinal covariates have been proposed that reduce the dimensionality of the covariates and subsequently apply RSF to dynamically predict the event [24, 25]. In our proposal, RSFs fit at each landmark time can incorporate updated longitudinal information in survival prediction while requiring no assumptions about the relationship between the longitudinal trajectory and the survival process.

We aim to assess the utility of using RSF in a landmarking framework as an approach for dynamic prediction of time-to-event outcomes using longitudinal biomarkers. A simulation study is performed comparing the predictive performance of a joint model, a Cox landmarking model and the proposed RSF landmark approach under scenarios of non-proportional hazards and model misspecification as well as inclusion of multiple longitudinal markers. In an application to dynamically predicting death after heart valve transplant, we compare the predictive performance of different model-building decisions for RSF landmarking. Additionally, we illustrate the use of RSF landmarking to obtain dynamic predictions for the development of de novo Donor Specific Antibodies (dnDSA) using longitudinal measurements of the immunosuppression drug Tacrolimus (TAC) in kidney transplant patients.

The structure of the paper is as follows. In Section “Methods”, we describe the extension of RSF to be used in a landmarking framework and give details about how to assess the prediction accuracy. Sections “Simulation study” and “Application” assess the methods via simulation and data application, respectively. The “Discussion” section includes concluding remarks as well as future directions, followed by “Conclusions” which includes brief recommendations.

## Methods

### Dynamic predictions

The observed data is given by *D*_*n*_={*T*_*i*_,*δ*_*i*_,*x*_*i*_,*y*_*i*_;*i*=1,...*n*} where $T_{i} = \min (T_{i}^{*}, C_{i})$ is the observed event time for the *i*-th subject (*i*=1,...,*n*), with $T_{i}^{*}$ denoting the true event time, *C*_*i*_ is the censoring time, and $\delta _{i} = I(T_{i}^{*} \leq C_{i})$ the event indicator. We observe *x*_*i*_, the baseline covariate vector, in addition to *y*_*i*_, a continuous vector of the longitudinal measurements for the marker, with *y*_*ij*_=*y*_*i*_(*t*_*ij*_) denoting the value of the marker observed at time *t*_*ij*_ for *j*=1,...,*n*_*i*_, where *n*_*i*_ is the number of marker measurements for individual *i*.

We are interested in obtaining a predicted probability of survival for a new subject *m* from the same population given their history of longitudinal marker measurements and baseline covariate data. Specifically, the aim is to obtain the predicted probability of surviving to a prediction horizon *τ*+*s,s*>0, given that subject *m* has survived up to landmark time *τ*, where *s* is a specified prediction window of interest. That is, our dynamic predictions are defined as:

1 $$ \begin{aligned} &\pi_{m}(\tau+s \mid \tau, x_{m}, \bar{y}_{m}(\tau))\\&= Pr(T_{m}^{*} \geq \tau + s \mid T_{m}^{*} > \tau, D_{n}, x_{m}, \bar{y}_{m}(\tau)).  \end{aligned}  $$

This prediction is conditional on a summary value of the history of the marker up to time $\tau, \bar {y}_{m}$. For the purposes of this manuscript, we assume that this summary measure is the scalar value of the last observation carried forward (LOCF), *y*_*m*_(*τ*). This formulation can be extended to include multiple longitudinally-collected markers.

### Landmarking

Landmarking involves selecting only the subset of subjects still event-free at landmark time *τ* and using a survival model to estimate the probability of surviving to the prediction horizon *τ*+*s*, where the prediction window *s* is pre-specified. There is often interest in many landmark times *τ*_1_,*τ*_2_,…,*τ*_*L*_, and thus landmarking involves developing a prediction model at each time. We construct a prediction data set for each landmark time *τ*_*l*_ for the risk set *R*(*τ*_*l*_)={*i*:*T*_*i*_>*τ*_*l*_}, consisting of individuals still event-free and uncensored at time *τ*_*l*_. To reduce the potential for bias that can occur from violation of the proportional hazards assumption within a prediction interval, administrative censoring is applied to the individuals’ event times at the pre-specified prediction horizon, *τ*_*l*_+*s* [6, 7]. The selection of multiple landmark times allows the same subject to contribute to the estimation of many predicted residual time distributions. For a model to be fit at each landmark time, every subject still in the risk set must have a value for the longitudinal marker at that landmark time. If the marker is not continuously observed, this value may not be available in practice and is imputed, most commonly using LOCF [6]. A separate survival model is then fit at each *τ*_*l*_ to obtain an estimate of the landmark-specific effect of the marker for predicting survival between *τ* and the fixed horizon *τ*+*s* for the risk set *R*(*τ*). For Cox landmarking, the proportional hazards model at each landmark time *τ* is given by *h*(*t*|*τ*,*x,y*(*τ*))=*h*_0,*τ*_(*t*) exp{*x*^′^*ξ*_1,*τ*_+*y*(*τ*)*ξ*_2,*τ*_} for *τ*≤*t*≤*t*+*s*, where *h*_0,*τ*_(*t*) is the landmark-specific baseline hazard function and *ξ*_*τ*_=(*ξ*_1,*τ*_,*ξ*_2,*τ*_)^′^ is the vector of landmark-specific coefficient estimates corresponding to that landmark time.

### Random survival forests

Random Survival Forests is an ensemble tree-based method for the analysis of right-censored survival data and is an extension of the random forest method [18, 26]. Survival trees are built by recursively partitioning the covariate space using binary splits to form groups of subjects who are similar according to the survival outcome [27]. The RSF predictor ensemble is formed by aggregating the results of many survival trees. Here, we present the RSF algorithm for creating a tree ensemble for predicting survival for a given set of covariates, as described in [28].

1. Draw *B* (*ntree*) subsamples of a specified sample size from the original dataset (with or without replacement).
2. Grow a survival tree for each subsample *b*=1,...,*B*.
  - At each tree node select a subset of the predictor variables available to try as candidates for splitting (*mtry*).
  - Select the node split from the candidate variables that maximizes the survival difference between the daughter nodes based on selected split criterion (*splitrule*) up to the number of predefined split points (*nsplit*).
  - Repeat (a)-(b) recursively on each daughter node until stopping criterion is met, often the number of unique cases in each terminal node (*nodesize*).
3. Calculate the cumulative hazard function for each tree using the terminal nodes. Aggregate information from the *B* survival trees to obtain a risk prediction ensemble.

This method is implemented in the R package **randomForestSRC** with the function *rfsrc()* and can be tailored based on different ensemble parameters that affect tree building [29]. Selection of these parameters can change the results of the predictions [29], and in Table 1 we show the inputs used in this manuscript [26]. The parameters that affect each node split include *mtry* and *nsplit*, which allow selection of how many variables should be included as candidates for splitting and how many splits should be considered for each candidate variable respectively. Specifying the number of splits considered for each candidate variable can reduce computation time compared to testing all possible split points for each covariate. The parameter *nodesize* denotes the number of unique cases in each terminal node and is mentioned in the existing software package documentation as something that should be experimented with to improve predictive performance.

**Table 1:** Parameters of a Random Survival Forest with suggested values

Parameter	Description	Suggested value
*ntree*	how many subsamples should be performed	500-1000
*sampsize*	proportion of the data should be selected for each subsample	63% of available data
*samptype*	Sampling Done With Replacement (swr) or without replacement (swor)	swr
*nodesize*	number of unique cases in each terminal node	15
*mtry*	how many variables should be included as candidates for splitting	$\sqrt {\text {\# variables}}$
*nsplit*	how many splits should be considered for each candidate variable	10
*splitrule*	impurity measure which aims to create the two most dissimilar daughter nodes at each split	“logrank”

### RSF ensemble for dynamic prediction

At each landmark time, the general RSF algorithm is applied to the corresponding landmark data set to build a risk prediction ensemble. Specifically, for landmark time *τ* we apply the RSF algorithm to the risk set *R*(*τ*)={*i*:*T*_*i*_>*τ*} using predictor variables $K_{i}=(x_{i}, \bar {y}_{i}(\tau))$ for each subject *i* in the risk set. Once each tree has reached the stopping criteria of the selected split type, the ensemble construction consists of aggregating tree-based Nelson-Aalen estimators. We extend the Morgensen et. al [28] formulation to our landmark framework. For each subsample *b* drawn from risk set *R*(*τ*), we obtain a survival tree, denoted $Q^{\tau }_{b}$ and a unique terminal node of subjects with the covariate vector $K=(x,\bar {y}(\tau))$, denoted $Q^{\tau }_{b}(K)$. Let $c^{\tau }_{{ib}}$ be the number of times that subject *i* is selected in the *b*th subsample for landmark time *τ*. For subject *i*, we define the counting process as *N*_*i*_(*t*)=*I*(*T*_*i*_≤*t*) and the at-risk process as *Y*_*i*_(*t*)=*I*(*T*_*i*_>*t*) [30]. For landmark time *τ*, we then define the landmark counting process and at-risk processes as $N^{\tau }_{i}(t)=I(T_{i}\geq \tau)N_{i}(t)$ and $Y^{\tau }_{i}(t)=I(T_{i}\geq \tau)Y_{i}(t)$, respectively. Then, using counting process notation as in [28], we define the aggregated counting and at-risk processes for each subsample *b* based on the landmark population, respectively, as

$$\begin{array}{*{20}l} N_{b}^{\tau}(t,K) &= \sum_{i = 1}^{n} c^{\tau}_{{ib}} I(K_{i} \in Q^{\tau}_{b}(K)) N^{\tau}_{i}(t); \\ Y_{b}^{\tau}(t, K) &= \sum_{i = 1}^{n} c^{\tau}_{{ib}} I(K_{i} \in Q^{\tau}_{b}(K)) Y^{\tau}_{i}(t). \end{array} $$

where in the terminal node corresponding to covariate vector *K*, $Y_{b}^{\tau }(t,K)$ is the number at risk at time *t* and $N_{b}^{\tau }(t, K)$ is the number of uncensored events up to time *t*. To estimate the conditional cumulative hazard $H_{b}^{\tau }(t|\tau,K)$ for the terminal node corresponding to covariate vector *K* in the *b*th subsample, we use a nonparametric conditional Nelson-Aalen estimator [31] that is based on subjects at risk at time *τ* and is given by

$$\hat{H}^{\tau}_{b}(t \mid K) = \int_{\tau}^{t} \frac{N_{b}^{\tau}(du,K)}{Y_{b}^{\tau}(u, K)}. $$

The dynamic prediction for a new patient with covariate vector $K_{m}=(x_{m},\bar {y}_{m})$ at prediction time *τ* as defined in Eq. (1) is then given by

$$\hat{\pi}_{m}(\tau+s|\tau,K_{m}) = \exp \left\{ -\frac{1}{B} \sum_{b=1}^{B} \hat{H}^{\tau}_{b}(\tau + s \mid K_{m}) \right\}. $$

### Additional considerations for building RSF models

Since the landmark formulation involves fitting a new ensemble at each landmark time *τ*, special consideration of the data and model formulation can occur at each prediction time. Instead of excluding individuals with missing baseline predictors, since we are using a tree-based algorithm, a forest can be grown in RSF to impute missing values to improve predictions [26]. This process can be done both when building the predictive risk ensembles, or when obtaining predictions for new individuals. In addition, the user-specified tuning parameters for the landmark-specific RSF can be adjusted separately at each landmark time to achieve optimal predictive performance. The function *tune.rfsrc()* is available in the **randomForestSRC** package and uses grid search to determine the optimal terminal node size (*nodesize*) and number of variables to try as candidates for splitting (*mtry*) [29]. The optimal values for these parameters are selected to minimize the out-of-bag (OOB) prediction error, which is estimated using Harrell’s concordance index [26]. Tuning is performed using fast trees, which builds survival forests using the specified number of subsamples and sampling type. This reduces computational speed but may not be as accurate as using the full forest [29].

### Variable importance

RSFs can capture nonlinear relationships between multiple covariates for predicting survival, and the landmarking RSF allows for these relationships to change over time. RSF gives an interpretation of variables that play a key role in predicting the survival outcome by providing a Variable Importance (VIMP) measure for each predictor. VIMP is defined as the change in prediction error on a new test case if variable *x* were not available, given *x* was used to grow the original forest. This is calculated via permutation of out-of-sample (OOB) observations that were not involved in building the trees [12, 32]. VIMP is a relative measure compared to the other variables included in building the forest ensemble. A zero or negative VIMP value at a particular landmark time reflects that the variable does not contribute predictive power for surviving the prediction window at that prediction time during a patient’s follow-up. VIMP can change during follow-up for the different covariates and can be used by clinicians to identify how the importance of the covariates change as the patient survives to time points beyond baseline.

### Assessing predictive performance

We assess the predictive performance of each method based on calibration and discrimination. Area under the receiver operating characteristic curve (AUC) was used to assess discrimination, and Brier score (BS) as an overall measure for both calibration and discrimination, and root mean square prediction error (RMSPE) was used to compare predicted probabilities to the true survival probabilities. To account for the censoring present in a survival outcome as well as the changing incidence at each landmark time, we use time-dependent versions of these measures [33]. The time-dependent AUC, *AUC*(*τ*,*s*), is used to determine how well the predicted probabilities discriminate between those likely and unlikely to experience an event in the prediction window *s* given that they are event-free at landmark time *τ*. This value ranges from 0 to 1, with higher values indicating better discrimination. The dynamic Brier score, *BS*(*τ*,*s*), is defined as the mean square error between the true event indicator and the predicted probability of an event in the prediction window *s* for those event-free at landmark time *τ* [34], with lower values indicating better predictive ability.

## Simulation study

A simulation study was performed to compare the predictive performance of the RSF landmarking approach to a joint model and Cox landmarking method. Specifically, the four main aims were to compare performance under the scenarios of (1) a simple relationship between the longitudinal and survival processes, (2) a violation of the proportional hazard assumption and a complex dependence of the longitudinal marker process, (3) multiple longitudinal outcomes, and (4), multiple noise variables not associated with the survival process.

### Data generation

The data were generated using a joint model. The longitudinal processes for *k* longitudinal outcomes were modeled using a linear mixed effects model that included only main effects for three binary baseline covariates and a random intercept and slope.

$$y_{{ik}}(t) = m_{{ik}}(t) + \epsilon_{{ik}}(t) = x_{{ik}}' \beta_{k} + z_{{ik}}' b_{{ik}} + \epsilon_{{ik}}(t) $$

where the longitudinal measurements *y*_*ijk*_={*y*_*ik*_(*t*_*ij*_),*j*=1,…,*n*_*ik*_} are simulated for each subject *i* for measurement time *t*_*ijk*_, and *x*_*ik*_ and *z*_*ik*_ are covariate vectors associated, respectively, with the vector of fixed effects *β*_*k*_ and vector of random effects *b*_*ik*_,*b*_*ik*_∼*N*(0,*G*_*k*_), where *G*_*k*_ is the variance matrix, and is independent of $\epsilon _{{ik}}(t)\sim N(0,\sigma _{k}^{2})$. The estimate of true unobserved value of each underlying longitudinal covariate is *m*_*ik*_(*t*). In this simulation study, we simulate two longitudinal markers, *m*_*i*1_(*t*) and *m*_*i*2_(*t*), where *x*_*i*1_=*x*_*i*2_=(1,*t,X*_1_,*X*_2_,*X*_3_)^′^ and *z*_*i*1_=*z*_*i*2_=(1,*t*)^′^.

For Aims 1-2 including only one longitudinal outcome, two different scenarios were assumed for the survival process, each with varying complexity in the association between the survival and longitudinal processes. In Scenario I, we simulated from a standard joint model where the hazard of the event depended only on the true current marker level. Scenario II included an interaction between log(1+*t*) and the association parameter to induce a violation of proportional hazards, and also included a quadratic trend in the relationship between the longitudinal marker and the survival outcome:

$$\begin{array}{*{20}l} {}\text{Scenario I: }& h_{i}(t) = h_{0}(t)\exp\{ \gamma'w_{i} + \alpha_{1} m_{i1}(t)\}. \\ {}\text{Scenario II: }& h_{i}(t) = h_{0}(t)\exp\{ \gamma'w_{i} + \alpha_{1} m_{i1}(t)^{2}\log(1+t)\} \end{array} $$

where *w*_*i*_=(*X*_*i*1_,*X*_*i*2_,*X*_*i*3_)^′^ is the vector of baseline covariates associated with the vector of coefficients *γ*=(*γ*_1_,*γ*_2_,*γ*_3_)^′^. The scalar *α*_1_ quantifies the association between the error free current measurement, *m*_*i*1_(*t*), and the time-to-event outcome. We use a Weibull baseline hazard, $\phantom {\dot {i}\!}h_{0}(t) = \exp (\sigma _{1}) \sigma _{2} t^{\sigma _{2}-1}$.

For Aim 3, we included the second longitudinal marker using a quadratic relationship with the marker and the survival process:

$$\begin{array}{*{20}l} {}\text{Scenario III: }& h_{i}(t) \,=\, h_{0}(t)\exp\{ \gamma'w_{i} \,+\, \alpha_{1} m_{i1}(t) \,+\, \alpha_{2} m_{i2}(t)^{2} \}. \\ \end{array} $$

A total of 500 simulated data sets of 1000 subjects each were generated. The subjects were assumed to have been followed for a period of up to 15 years, with longitudinal measurements at baseline and at up to 30 follow-up times generated from an exponential distribution with a rate of 2 per year for both longitudinal outcomes. Censoring was simulated under a uniform distribution. Each of the scenarios resulted in approximately 50%, 43%, and 45% censoring, respectively. To address the fourth aim of the simulation study, an additional set of 7 binary “noise” variables, *X*_*i*4_,…,*X*_*i*10_, were simulated with varying prevalence, but not included in generating the survival and longitudinal data under Scenarios I, II, or III. Additional details and the parameter values for the simulation are given in Supplementary Section 1 in Additional file 1.

### Model fitting

An identical procedure was repeated on data sets from Scenario I, II and III. Each dataset was randomly split into 50% training and 50% testing data sets. Each prediction method was then fit to the training sets and used to compute dynamic survival probabilities on the 500 test individuals, which we used to assess predictive performance. Evaluation of predictive ability via AUC, Brier score and RMSPE was performed at five follow-up times for each scenario, and values were averaged across simulated data sets. A prediction window of *s*=5 years was used for all three scenarios.

We fit a joint model as specified in data generation with a linear mixed effects model for the longitudinal process and the true current value of the marker included in the survival process. This model was correctly specified for Scenario I, but misspecified for Scenario II. For Scenario III, we fit two misspecified joint models. One in which fit the longitudinal marker *y*_1_ as in Scenarios I and II, and the longitudinal marker *y*_2_ is treated as a baseline covariate, and a second in which we include both longitudinal markers using a multivariate shared parameter joint model for longitudinal and survival outcomes under a Bayesian estimation approach (implemented using the **JMBayes** R package), ignoring the quadratic relationship with the hazard. We used Cox landmarking by fitting a simple Cox model with LOCF imputation of the longitudinal marker at each of the landmark times. Details of the Cox model and joint model that were fit and how dynamic predictions were obtained can be found in Supplementary Section 2 (Additional file 1).

For the RSF landmarking approach we fit a RSF at each landmark time, using LOCF for the longitudinal marker as in the Cox landmarking. Each RSF was run with 1000 trees and all other suggested values in Table 1. We also fit the RSF models with parameter tuning at each landmark time to select the number of covariates to try as candidates (*mtry*) and final node size. We fit all the modeling approaches twice: (1) with *w* including only the 3 baseline variables that were used in the data generation, as well as (2) including the 7 simulated noise variables.

### Results

**Fig. 1:**
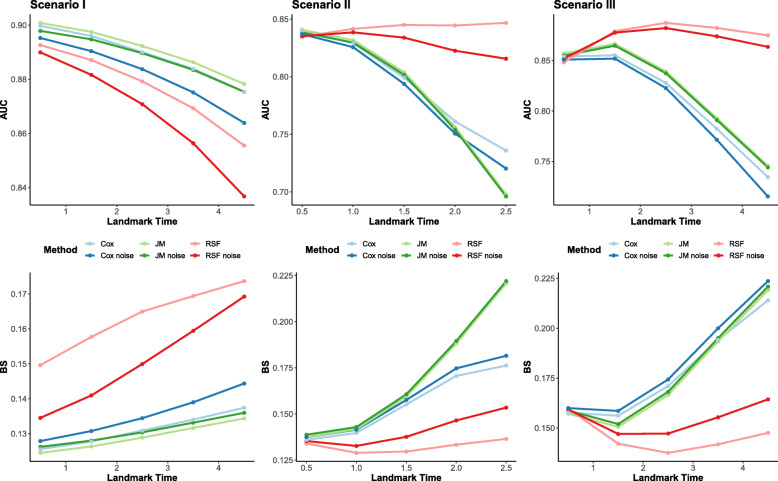
Simulation Results. Simulation estimates for AUC (upper panels) and BS (lower panels) for predicted probability *P*(*T*^∗^≤*τ*+5∣*T*^∗^>*τ*,*y*(*τ*),*x*) from a Cox landmarking model (Cox), joint model (JM), and RSF landmarking approach (RSF). Left panels: a linear trend in marker (Scenario I), middle panels: quadratic trend in marker with non proportional hazards (Scenario II), right panels: Inclusion of two longitudinal covariates (Scenario III, joint model shown is the multivariate model). The lighter “noise” lines represent the models including the additional 7 simulated variables that were not used in generating longitudinal and survival data

In Scenario I (Fig. 1, left panel), when there is a simple relationship between the survival and marker processes where the survival depends only on the true current level of the marker, the RSF landmarking approach does not perform better than the Cox landmarking or correctly specified joint model based on both AUC and Brier score. The RMSPE followed a similar pattern to BS (Additional file 1 Table S3) The AUC of all models decrease over time, which is likely due to a selection process that induces increasing homogeneity in the at-risk population at later prediction time points [33]. The landmark Cox and RSF models experience a greater decrease in AUC over time possibly since these models are trained on smaller data sets at later prediction times.

In Scenario II (Fig. 1, middle panel), when we violate the proportional hazards assumption and introduce a more complex relationship between the marker and the survival process, RSF landmarking performed better in terms of both AUC and BS at every time point except the earliest landmark where it performed similarly to the Cox landmarking approach and the misspecified joint model. The performance of RSF landmarking was similar across all landmark times. In contrast, both the misspecified joint model and Cox landmarking had worsening predictive performance at later landmark times, with the largest decrease over time seen in the misspecified joint model. The RMSPE of the models can be seen in Additional File 1 Table S6, and follows a similar pattern to BS.

In Scenario III (Additional File Tables S7-9), the naïve joint model that uses only longitudinal information from one marker performed similarly to RSF at baseline, but at later landmarks had worse performance. The multivariate joint model that uses both longitudinal markers (Fig. 1, right panel) performed slightly better than RSF at early landmark times but followed the same pattern of reduction over time as the naïve joint model. The Cox landmarking approach had performance similar to the joint models at later landmark times as the longitudinal quadratic relationship between *y*_2_ and the hazard changed. The RSF method performed consistently across time points and was closest to the true probability at every landmark time, with AUC, BS, and RMSPE showing similar patterns.

In all scenarios, all methods (Cox noise, JM noise, RSF noise) had decreased performance when variables were included that were not associated with the survival process (Fig. 1). In Scenario I (simple relationship), the performance of the RSF decreased more with the inclusion of the noise variables compared to the other two methods. In Scenario II (complex relationship), the reduction in the RSF landmark and joint modeling approach was less severe than the Cox landmark approach. All predictions were similar with and without noise in Scenario III. In Figures S1 in Additional file 1, we demonstrate that for Scenario II the VIMP of the noise variables under the RSF landmarking approach is close to 0 across the landmark times.

Estimation and prediction using the Cox landmarking model, the joint model, and RSF landmarking took on average about 25 s (s), 80s, and 19s, respectively, for the 5 landmark times for all scenarios. The multivariate joint model took much longer at about 15 min on average. When noise covariates were included, estimation and prediction of the RSF landmarking increased to around 30s. RSF landmarking with tuning increased time by about 10s for the correct set of covariates and about 30s with the noise variables included. No substantial improvements in predictive performance were seen from tuning in Scenario I and across all scenarios when no noise is present. But, with the more complex relationships in Scenarios II and III, slight improvements in AUC, BS and RMSPE can be seen in the tuned compared to author-selected parameter models when noise variables are included (Additional File 1, Tables S1-S9).

Overall, from the results of our simulation study we found that (1) RSF landmarking did not have good predictive performance when there was a simple relationship between the survival and longitudinal marker processes and few baseline covariates; (2) When the relationship between the marker and survival process was complex, and the proportional hazards assumption violated, the RSF landmark approach performed better than Cox landmarking and a misspecified, simple joint model; (3) The RSF method can accommodate more than one longitudinal covariate with good predictive performance relative to misspecified joint modeling and Cox landmarking; (4) When extra noise variables are included and there are complex relationships present, including these covariates in the RSF landmarking reduces predictive performance only slightly; (5) Parameter tuning when predicting with a small number of covariates did not substantially improve performance and may not be necessary.

## Application

We use two data applications to demonstrate (1) the predictive performance of various RSF model specifications, and (2) the computation and use of variable importance and individualized dynamic predictions from a RSF landmarking approach.

### Heart valve transplant

We use an observational study of patients who received an aortic valve replacement surgery [35] to assess the predictive performance of RSF landmarking and evaluate various model specifications in a clinical application. Data were available for 256 patients who were followed post surgery for whom longitudinal measurements were collected for two different heart function measures, left ventricular mass index (LVMI) and ejection fraction. Data are publicly available in the **joineR** package in R software [36]. The aim was to use these longitudinal marker measurements and baseline patient characteristics to predict the probability of surviving a prediction window of 3 years at landmark times 0.5, 1, 1.5, 2, 2.5, 3 years post surgery.

We fit several variations of the RSF landmarking approach to evaluate various RSF modeling specifications: imputation, administrative censoring, tuning, and variable selection (Additional file 1, Table S10). We compare the predictive performance of these RSF landmark models to each other, as well as to Cox landmarking, and a joint model for survival and LVMI that assumes a longitudinal marker trajectory for LVMI, and a dependence structure based on the true current LVMI level. All models included the following clinically relevant variables: sex, age, preoperative left ventricular ejection fraction, presence of concomitant coronary artery bypass graft, and implanted aortic prosthesis type. We also fit RSF models with a larger set of 16 baseline predictors. The longitudinal marker of ejection fraction was included in the RSF and Cox models, but excluded from the joint model due to the associated increase in modelling complexity. For both the RSF and Cox landmarking, the longitudinal measures were imputed at landmark times using two different approaches, LOCF and subject-specific predictions from a linear mixed model (LMM). Predictive performance measures of BS and AUC, as described previously, were computed at each landmark time using 5-fold cross-validation, repeated and averaged across 100 iterations.

First, we compare the RSF landmark approach with Cox landmarking and joint modeling (Additional file 1, Figure S2). The RSF approach has similar predictive performance to Cox landmarking in terms of AUC and BS, with the RSF models having slightly higher AUC and lower BS at earlier landmark times. The RSF model has similar BS to the joint model at earlier landmark times but worse AUC, indicating that although the model has worse discrimination it is possibly better calibrated at earlier times.

**Fig. 2:**
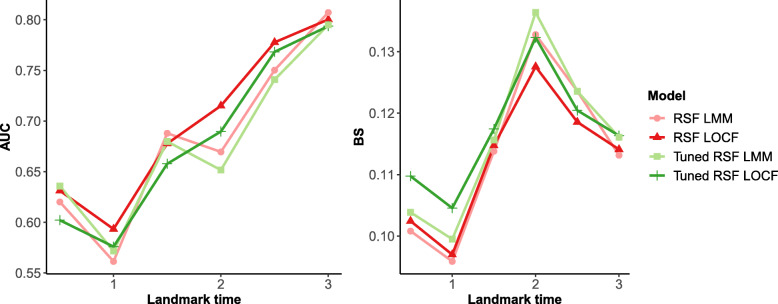
Heart Valve Application Results. Comparison of average cross-validated AUC (left panel) and BS (right panel) of RSF landmarking based on imputation method for missing longitudinal values. Tuned = RSF parameters were tuned; LOCF = imputation using last-observation-carried forward; LMM = imputation using subject-specific prediction from a mixed model for the longitudinal marker

Second, comparing within the various RSF specifications, we assess RSF models that use LOCF versus LMM imputation (Fig. 2). Based on AUC and BS, both the tuned and selected parameter RSF models that used the LOCF imputation performed better than their LMM imputed counterparts at later landmark times, but similarly or worse at earlier landmark times.

Third, as a nonparametric method, RSF does not rely heavily on the proportional hazards assumption, and thus the administrative censoring of the event time that is used in Cox landmarking may not be necessary. RSF landmarking without administrative censoring had similar, but slightly worse, predictive performance compared to the method using administrative censoring (Additional file 1, Figure S3).

Fourth, in high-dimensional settings, Cox and joint models can be restrictive in the number of variables that they can include for model stability. We compare a RSF model with a clinically-relevant set of variables to RSF landmark models that uses all of the 15 available baseline variables. AUC and BS were very similar for both methods, with the RSF models using all variables having slightly higher AUC at some landmark times (Additional file 1, Figure S4).

Fifth, we assess the performance of RSF for dynamic prediction using tuning to optimize the inputs for the number of variables to try at each node (*mtry*) and terminal node size. We found that tuned RSFs have worse performance in terms of AUC and BS compared to the selected settings given in Table 1 (Fig. 2, Additional file 1 Figure S4). This was seen in both the full and reduced models, as well as with both types of imputation.

Overall, we found that RSF shows good performance using the suggested parameter inputs and can allow for increased prediction abilities by imputing missing data within the RSF procedure without significantly affecting performance. In this particular data application, we find that the RSF models perform similarly to Cox landmarking, but not as well as the simple joint model. This is possibly due to the lack of complexity in the relationships between variables, as was explored in the simulation, or due to the limited sample size on which to build the RSF models.

### Kidney transplant study

To demonstrate RSF landmarking for computing individualized dynamic predictions, we use a retrospective study conducted at the University of Colorado Hospital which followed patients post-kidney transplant for up to 7 years. Data collected included basic baseline patient characteristics, as well as longitudinal immunosuppression drug levels (Tacrolimus, TAC), and the timing of the development of de novo Donor Specific Antibodies (dnDSA), an early warning signs of adverse outcomes. Additional study details can be found elsewhere [37, 38]. The objective was to determine an individual patient’s risk of developing dnDSA within 1 year using baseline factors and a patient’s up-to-date longitudinal history of TAC. With this application, we demonstrate the assessment of RSF variable importance (VIMP), and illustrate computation of patient-specific dynamic predictions of dnDSA using baseline and TAC data.

**Fig. 3:**
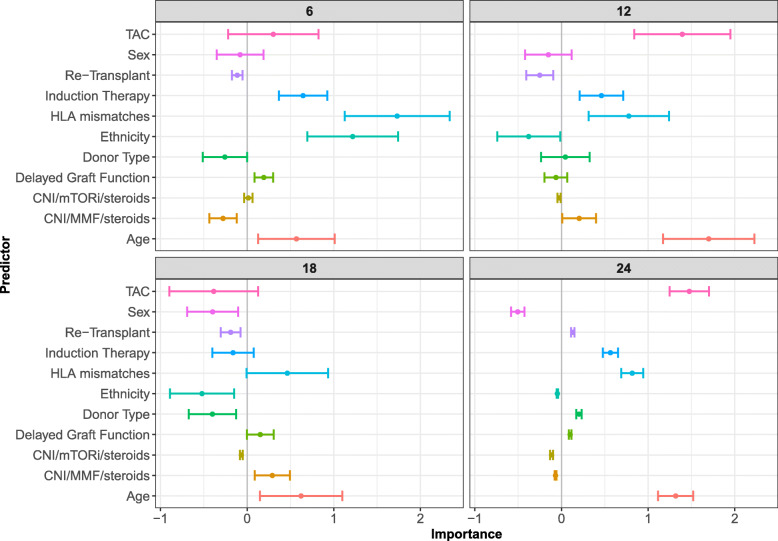
TAC Landmark VIMPs. Delete-*d* jackknife 95% asymptotic normal confidence intervals for VIMP for the Kidney Transplant data set at landmark times 6, 12, 18, and 24 months. Variables included in analysis are listed in the key. Donor Type = Deceased or Living donor; HLA mismatches = of mismatches between the kidney donor and kidney recipient; Induction Therapy = thymoglobulin or other; TAC = LOCF of TAC measurement

There were 535 individuals that met the final inclusion criteria, of which 178 (33%) experienced dnDSA during follow-up. Post transplant, patients were monitored for up to 7 years with up to 90 measures (median 22 [IQR: 15-32]) of TAC ranging from 0 to 30. Ten baseline covariates were included, the full list is given in Fig. 3.

We fit a RSF landmark model at each of the landmark times including all available covariates. The history of longitudinal TAC was summarized at each landmark time as a scalar value using LOCF. The repeated 5-fold average cross-validated AUCs at landmark times 6,12,18,24 months were 0.58, 0.57, 0.52, and 0.64, respectively.

Variable importance assessments were performed at landmark times *τ*= 6, 12, 18, 24 months with a prediction window of *s*=12 months (Fig. 3). VIMP confidence intervals were obtained using a delete-*d* jackknife estimator, that was calculated using 1000 subsamples [32]. The VIMP of TAC was variable across landmark times, but was generally becoming more important in predicting dnDSA as time from transplant increased, though reductions at landmark time 18 require additional investigation. While ethnicity was important early on, it had VIMP values below 0 for later times indicating that it may not be of much use predicting dnDSA when considerable time has lapsed since transplant. Age and the number of Human Leukocyte Antigens (HLA) mismatches proved to be relatively important at all timepoints and additional covariates to investigate include the type of induction and maintenance immunotherapy.

**Fig. 4:**
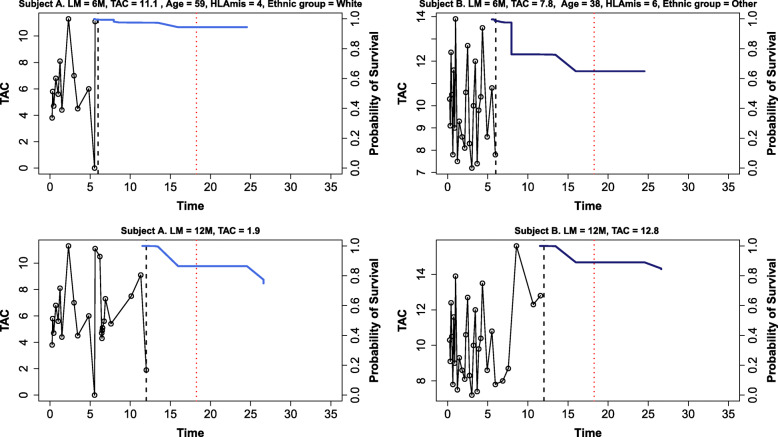
Individual Predictions. Longitudinal trajectory and RSF landmark conditional predicted probability of survival for s = 24 months at landmark times *τ*= 6 (top panels) and 12 (bottom panels) months for 2 individuals who experienced an event at *t*=18 months (red dotted lines). Model AUCs were 0.61 (*τ*=6) and 0.62 (*τ*=12). Black dotted lines represent transition from left axis of TAC values to right axis of predicted probability. HLAmis = HLA mismatches

As an example of dynamic prediction with RSF landmarking, we fit a reduced RSF landmark model including the covariates age, ethnic group, HLA mismatch, and last-observed TAC, that were shown to be important from the variable importance plot. We present the predicted conditional dnDSA-free survival curves for two individuals in the data set at landmark times of *τ*=6,12 months (Fig. 4). These two individuals had similar event times but differing baseline covariates. Subject A had a high TAC value at the first landmark time, while Subject B had a low TAC value, and Subject B’s survival curve is noticeably lower. Given that both individuals survive to 12 months, we update the survival predictions using their up-to-date TAC values 12 months post-transplant, and find that Patient B’s 12-month survival probability has improved based on their increased TAC value, indicating that changes in drug exposure can impact the development of dnDSA.

## Discussion

Dynamic prediction of survival outcomes is a powerful tool in making important clinical decisions. In this paper, we proposed a dynamic prediction method using a RSF landmarking approach. The use of a machine learning approach for dynamic prediction has practical and performance advantages over the existing methods of Cox landmarking and joint modeling.

In our simulation study, we assessed the performance of the RSF landmarking approach. When there are a small number of baseline variables and complex relationships do not exist between the marker and survival process, the RSF landmark approach does not have good predictive performance compared to Cox landmarking and a correctly specified joint model. However, when there was a complex relationship for the dependence between the marker process and the hazard and the proportional hazards assumption was violated, RSF landmarking outperformed a simple, misspecified joint model and Cox landmarking. In the setting with two longitudinal markers, the RSF landmarking approach, which does not require specification of the marker relationships in the survival process, outperformed the joint model that included both longitudinal markers but misspecified their relationship in the hazard function. As well, when there were noise variables included in the modeling that were not used in the data generation process, the predictive performance of all methods worsened, but the decrease in performance was less for the RSF landmark models than Cox landmarking. Thus, RSF landmarking is beneficial in situations in which there are complex relationships present between the marker and survival outcomes and when there are multiple predictors present, but their clinical relevance and predictive power is unknown.

In the heart valve transplant application, we assess the predictive performance of RSF landmarking and its various specifications in a clinical setting. We compared the imputation of the longitudinal markers in RSF landmarking using a LMM or LOCF, and found that with increasing landmark time the LOCF imputation had better predictive performance. This is possibly due to a misspecified LMM for the longitudinal markers that produces biased predictions at later landmark times. RSF with administrative censoring, which is used in Cox landmarking due to its reliance on the proportional hazards assumption, had slightly better predictive performance for the nonparametric RSF landmark models. Tuning of the RSF parameters increased computation time substantially but often did not improve predictive performance compared to using suggested parameters. This would indicate that the application of the default *tune.rfsrc*, which is based on Harrell’s concordance index, may not be sufficient in this context. While this measure is related to the time-dependent AUC [39], it may not be appropriate for assessing predictive performance for a fixed prediction horizon [40]. Instead, we can consider coding the selection of optimal tuning parameters that maximizes the time-dependent AUC metric at each landmark time. Selecting a small subset of clinically relevant variables compared to including all available variables did not improve performance consistently across landmark times. Overall, using RSF landmarking and LOCF imputation with suggested specifications was found to be sufficient for maintaining predictive performance and did not pose a computational burden.

In the kidney transplant application, we demonstrate the use of RSF landmarking for obtaining and understanding dynamic predictions to answer clinical hypotheses. Previous studies have found that age, ethnicity, HLA mismatches, and Tacrolimus were associated with development of dnDSA [37]. This was confirmed with the use of VIMP plots that also identified the novel predictor of type of maintenance immunosuppresion. Ethnicity was important in avoiding dnDSA immediately following transplant, but less so as time progressed post-transplant. Thus, the use of VIMP plots demonstrates how relationships between the survival outcome and predictors can change over time. This application also demonstrated how predictions of dnDSA can be updated in real time based on the TAC value at the landmark time. This provides a tool for personalized medicine, as it may help to inform the clinician whether to change the dosing of TAC based on the patient-specific risk of dnDSA.

Thus, an advantage of RSF landmarking is that this nonparametric, machine learning approach allows for us to capture complex, nonlinear relationships between the longitudinal and baseline variables in the hazard of the survival outcome. It does not rely on a proportional hazards assumption as in Cox landmarking, and does not require prior correct specification of the longitudinal trajectory of the marker or the dependence relationship between the marker and survival process as in joint modeling. As well, it has good predictive performance in the presence of noise variables, indicating that it is a useful approach when there are multiple predictors for which the clinical relevance is unknown. RSF landmarking is also beneficial in situations where multiple longitudinal biomarkers are present, as joint models require the specification of the longitudinal trajectory and their dependence in the hazard for each marker process. VIMP can also be useful in this case to see the changing predictive power of the markers over time. In addition, RSF landmarking is easily implementable in standard software and using specifications suggested in this manuscript requires less computation time than joint modeling, especially when there is more than one longitudinal marker or complex covariate relationships exist.

A limitation of RSF landmarking is that the predictions are not linked over time due to the use of independent RSF models at each landmark time results. Cox landmarking with landmark-specific baseline hazards and covariate effects suffers from the same issue, and thus both approaches do not provide a consistent prediction model [41]. Extensions to Cox landmarking include specifying the coefficient estimates and baseline hazard as a smooth function of landmark time to link the landmark models. To apply a similar smoothing technique in an RSF landmarking frameworks, we can compute the cumulative hazard used in the survival prediction at time *τ* as an average of the cumulative hazards for a sequence of landmark times surrounding *τ*. A similar approach was used for dynamic prediction with large scale data, where a sliding window in combination with RSF was used to put more emphasis on the recent versus older past [42].

In this work, we present a comparison of RSF landmarking and its various specifications to two existing methods and demonstrate situations in which it can be expected to have greater utility and predictive performance. Future work will require exploring the use of RSF landmarking in various situations for which extensions to RSFs exist but should be assessed before used in a landmarking framework, such as competing risks [13] and interval censored survival outcomes [43].Additionally, a comparison of our RSF approach to recently developed conditional inference and relative risk forests [44] for time-varying covariates is of interest. We have demonstrated the use of RSF landmarking using models built using the log-rank split rule that is based on a proportional hazards assumption [27]. Although, we did not find that this method had poor performance in situations where the proportional hazards assumption was violated, the use of other survival ensemble methods that use other splitting rules, such as conditional inference forests can be explored [45]. Additionally, as in Cox landmarking, we use a LOCF imputation for the longitudinal marker at landmark times at which it is not observed. We extend this in the heart valve data application where we explore the use of a model for the longitudinal marker to impute the marker value, as in [46]. However, alternative specifications can be considered using other summary measures such as the marker slope, or using a function of multiple time-varying covariates to grow trees [24, 25]. The ability of RSFs to deal with correlation among its predictors allows for us to include and evaluate multiple marker specifications when building the predictive model. Additional studies are required to demonstrate the use of RSF landmarking in high-dimensional settings.

## Conclusions

RSF landmarking is a nonparametric, machine learning alternative for dynamic prediction that can capture complex, nonlinear relationships between the longitudinal and baseline variables in the hazard of the survival outcome. It is a useful approach when there are multiple candidate predictors for which the clinical relevance is unknown or in situations where multiple longitudinal biomarkers are present. Examining the VIMP over time can provide insight into the changing predictive power of the markers over time. In addition, RSF landmarking is easily implementable in standard software and using specifications suggested in this manuscript requires less computation time than joint modeling.

## Supplementary Information

**Additional file 1** Supplemental materials. This is a PDF of supplemental information including equations and figures referenced in text.

## Data Availability

The heart valve data set is publicly available in R package joineR. The TAC dataset is not publicly available but may be made available upon reasonable request. Code for simulation datasets as well as models for simulation and heart valve data are publicly available at https://github.com/picketka/rsf_landmarking.

## Abbreviations

TAC: Tacrolimus
dnDSA: de novo Donor Specific Antibody
RSF: Random survival forest
HLA: Human leucocyte antigen
AUC: Area under the curve
BS: Brier score
LOCF: Last observation carried forward
JM: Joint model
