# Supplementary material for: Random survival forests for dynamic predictions of a time-to-event outcome using a longitudinal biomarker
Source: BMC Med Res Methodol. 2021 Oct 17;21:216. doi: 10.1186/s12874-021-01375-x (PMC8520610; doi:10.1186/s12874-021-01375-x)
Supplement: Supplementary file 1 — Additional file 1 Supplemental materials. This is a PDF of supplemental information including equations and figures referenced in text. [file 12874_2021_1375_MOESM1_ESM.pdf]

Random Survival Forests for Dynamic Predictions of a Time-to-Event Outcome Using a Longitudinal Biomarker
Additional File 1

K. Pickett, K. Suresh, K. Campbell, S. Davis, E. Juarez-Colunga

August 4, 2021

1 Simulation Data Generation Details

Recall that data were generated according to the joint model:

$$y_{ik}(t) = m_i(t) + \epsilon_i(t) = \beta_{0k} + \beta_{1k} time + \beta_{2k} X_{1i} + \beta_{3k} X_{2i} + \beta_{4k} X_{3i} + b_{i0} + b_{i1} + \epsilon_i(t), k = 1, 2$$

$$\text{Scenario I + II: } h_i(t) = h_0(t) \exp\{\gamma_0 + \gamma_1 X_{1i} + \gamma_2 X_{2i} + \gamma_3 X_{3i} + \alpha_1 Y_i(t)\}$$

$$\text{Scenario III: } h_i(t) = h_0(t) \exp\{\gamma_0 + \gamma_1 X_{1i} + \gamma_2 1 X_{2i} + \gamma_3 X_{3i} + \alpha_1 m_{i1}(t) + \alpha_2 m_{i2}(t)^2\}$$

The Weibull baseline hazard had a shape parameter of $\sigma_2 = 2$ for all scenarios.The covariates were all created from binomial distributions as follows: $X_1 \sim bin(0.60), X_2 \sim bin(0.50), X_3 \sim bin(0.55)$. The additional 7 covariates that were not included in data generation were distributed as follows: $X_4 \sim bin(0.50), X_5 \sim bin(0.65), X_6 \sim bin(0.45), X_7 \sim bin(0.55), X_8 \sim bin(0.70), X_9 \sim bin(0.60), X_10 \sim bin(0.50)$

While Y_i differed between Scenario I and II and parameters were set as was shown in the data generation section, all other parameters were identical between scenarios and will be described below. The coefficients were defined as $bi = (b_{i0}, b_{i1} \sim N(0, G)$ with $G = \begin{pmatrix} 1 & 0.5 \\ 0.5 & 1 \end{pmatrix}$ and $\epsilon_i(t) \sim N(0, \sigma^2)$ with $\sigma^2 = 0.6$. The other coefficients were $\beta_{01} = 5.6, \beta_{11} = -0.45, \beta_{21} = -0.25, \beta_{31} = -0.11, \beta_{41} = -0.3$. The coefficients for the survival submodel were $\gamma_0 = -8, \gamma_1 = 1.9, \gamma_2 = 2.15, \gamma_3 = 2.65, \alpha_1 = 0.07$.

The coefficients for Scenario III were defined slightly differently. The coefficients for y_1 were same as the previous scenarios. The coefficients for y_2 were $\beta_{02} = 2.0, \beta_{12} = 0.25, \beta_{22} = 0.75, \beta_{32} = -0.61, \beta_{42} = 0.45$. The coefficients for the survival submodel were $\gamma_0 = -9, \gamma_1 = 1.9, \gamma_2 = 2.15, \gamma_3 = 2.65, \sigma_2^2 = 0.4, \alpha_1 = 0.09, \alpha_2 = 0.05$.

2 Comparison Models for Application

Joint Model Formulation and Prediction

A linear mixed effects model that included only main effects for the three binary covariates and time with a random intercept and slope as described in the simulation setup was fit:

$$y_i(t) = m_i(t) + \epsilon_i(t) = x_i'\beta + z_i'b_i + \epsilon_i(t).$$

The estimate of true unobserved value of the underlying longitudinal covariate, $m_i(t)$, was then used to quantify the effect on the risk of the event:

$$h_i(t \mid M_i(t), w_i) = h_0(t) \exp\{\gamma' w_i + \alpha m_i(t)\}$$

where $M_i(t) = \{m_i(u); 0 \leq u < t$ denotes the history of the true unobserved longitudinal process up to time t.

Following a Bayesian formulation of the joint model, the estimation of $\pi_m(t_{hor} \mid \tau)$ is based on the posterior predictive distribution:

$$\pi_m^{JM}(t_{hor} \mid \tau) = \int Pr(T_m^* \geq t_{hor} \mid T_m^* \geq \tau, y_m(t), \theta) p(\theta \mid D_n) d\theta,$$

where θ is the vector of all model parameters. The calculation of the first term requires the conditional independence assumption. The first term of the integrand of $\pi_m^{JM}(t_{hor} \mid \tau)$ can be written as

$$Pr(T_m^* \geq t_{hor} \mid T_m^* \geq \tau, Y_m(t), \theta) = \int Pr(T_m^* \geq t_{hor} \mid T_m^* \geq \tau, b_m, \theta) p(b_m \mid T_m^* \geq \tau, y_m(t), \theta) db_m =$$
$$= \int \frac{S_m\{t_{hor} \mid M_m(t_{hor}, b_m), \theta\}}{S_m\{\tau \mid M_m(\tau, b_m), \theta\}} p(b_m \mid T_m^* \geq \tau, y_m(t), \theta) db_m,$$

where

$$S_m\{\tau \mid M_m(\tau, b_m), \theta\} = \exp\left\{ \int_0^\tau h_0(s) \exp\{\gamma^T x_m + \alpha M_m(s)\right\} ds$$

The integral can be estimated by Monte Carlo sampling and a Metropolis-Hastings algorithm.

Cox Model Formulation and Prediction

As was briefly mentioned in the landmarking section, the Cox landmarking formulation hazard can is

$$h(t|\tau, x, y(\tau)) = h_{0,\tau}(t) \exp\{x' \xi_{1,\tau} + \tilde{y_i}(t) \xi_{2,\tau}\}; \tau \leq t \leq t + s$$

Where $h_{0,\tau}(t)$ is the landmark-specific baseline hazard function and $\xi_\tau = (\xi_{1,\tau}, \xi_{2,\tau})'$ is the vector of coefficient estimates corresponding to that landmark time and the dependence of the baseline hazard on τ is modeled by estimating a different baseline hazard for each τ, $h_0(t \mid \tau) = h_{0\tau}(t)$.

Once the Cox model was fit, the required residual time distribution, $\pi_m(\tau{+}s \mid \tau)$, was calculated for each new individual :

$$\hat{\pi}_m^{LM}(t_{hor} \mid \tau) = \exp[-\int_\tau^{\tau+s} h_i(u \mid \tau, y(\tau), \boldsymbol{x}) du],$$

Multivariate Joint Model Formulation and Prediction

The function *mvJointModelBayes* was used to fit a multivariate shared parameter joint model for the longitudinal and survival outcomes under a Bayesian approach in Scenario III. Both longitudinal markers were fit with a gaussian family in the *mvglmer* function. Predictions were obtained similarly to the joint model described above using average estimates from Monte Carlo simulations.

3 Additional Result Figures and Tables: Simulation

Table S1: AUC results from Simulation Scenario I

Landmark Time	True Model	JM	JM Noise	RSF tuned	RSF default	RSF noise tuned	RSF noise default	Cox	Cox Noise
0.5	0.9067	0.9008	0.8979	0.8908	0.8926	0.8886	0.89	0.8998	0.8953
1.5	0.9009	0.8975	0.8948	0.8858	0.8871	0.8799	0.8816	0.896	0.8904
2.5	0.8943	0.8923	0.8896	0.875	0.8792	0.8675	0.8708	0.89	0.8837
3.5	0.8881	0.8863	0.8835	0.8628	0.8693	0.8532	0.8564	0.8839	0.8752
4.5	0.8799	0.8783	0.8754	0.8488	0.8555	0.8324	0.8368	0.8754	0.8639

Table S2: BS results from Simulation Scenario I

Landmark Time	True Model	JM	JM Noise	RSF tuned	RSF default	RSF noise tuned	RSF noise default	Cox	Cox Noise
0.5	0.121	0.1245	0.1262	0.1456	0.1496	0.1454	0.1345	0.1256	0.1279
1.5	0.1241	0.1263	0.128	0.1543	0.1577	0.1476	0.1409	0.1278	0.1307
2.5	0.1273	0.1289	0.1304	0.1571	0.165	0.1488	0.1499	0.1309	0.1344
3.5	0.1299	0.1316	0.1332	0.1602	0.1694	0.1554	0.1594	0.134	0.139
4.5	0.1327	0.1344	0.136	0.1647	0.1737	0.1646	0.1693	0.1375	0.1444

Table S3: RMSE results from Simulation Scenario I

Landmark Time	JM	JM Noise	RSF tuned	RSF default	RSF noise tuned	RSF noise default	Cox	Cox Noise
0.5	0.0583	0.0707	0.1529	0.1695	0.1516	0.116	0.0662	0.0812
1.5	0.0485	0.0625	0.1699	0.1838	0.1474	0.1294	0.0603	0.0802
2.5	0.0418	0.0571	0.1685	0.1946	0.144	0.1503	0.0589	0.0831
3.5	0.04	0.0557	0.1707	0.1995	0.1568	0.1715	0.0613	0.091
4.5	0.0406	0.0565	0.1769	0.2026	0.1757	0.1904	0.0672	0.1045

Table S4: AUC results from Simulation Scenario II

Landmark Time	True Model	JM	JM Noise	RSF tuned	RSF default	RSF noise tuned	RSF noise default	Cox	Cox Noise
0.5	0.9639	0.8407	0.8384	0.8335	0.8346	0.8232	0.8352	0.8398	0.8369
1	0.9638	0.8318	0.8296	0.8349	0.8416	0.8271	0.8385	0.8291	0.8256
1.5	0.9622	0.8039	0.8017	0.8338	0.8449	0.8266	0.8339	0.7992	0.7938
2	0.9593	0.7564	0.7541	0.8333	0.8445	0.8255	0.8226	0.7612	0.7506
2.5	0.9575	0.6979	0.6961	0.8373	0.8467	0.8274	0.8156	0.7358	0.7203

Table S5: BS results from Simulation Scenario II

Landmark Time	True Model	JM	JM Noise	RSF tuned	RSF default	RSF noise tuned	RSF noise default	Cox	Cox Noise
0.5	0.0651	0.1376	0.1388	0.142	0.134	0.1393	0.1353	0.136	0.1352
1	0.0643	0.1418	0.1429	0.1378	0.1289	0.1351	0.1327	0.1397	0.1374
1.5	0.0665	0.1594	0.1607	0.1403	0.1296	0.1373	0.1376	0.1552	0.1491
2	0.07	0.1881	0.1895	0.1427	0.1334	0.1408	0.1465	0.1706	0.1653
2.5	0.0724	0.2208	0.222	0.1427	0.1365	0.144	0.1534	0.1762	0.178

Table S6: RMSE results from Simulation Scenario II

Landmark Time	JM	JM Noise	RSF tuned	RSF default	RSF noise tuned	RSF noise default	Cox	Cox Noise
0.5	0.2685	0.2707	0.277	0.2622	0.2647	0.2659	0.2653	0.2639
1	0.2777	0.2798	0.2712	0.2539	0.2612	0.2583	0.2743	0.27
1.5	0.304	0.306	0.2713	0.2507	0.266	0.2561	0.2968	0.2864
2	0.3422	0.3441	0.2681	0.2507	0.275	0.2567	0.3155	0.307
2.5	0.384	0.3855	0.2642	0.252	0.283	0.2576	0.3207	0.3236

Table S7: AUC results from Simulation Scenario III

Landmark Time	True Model	MV JM	MV JM Noise	JM naiive	JM naiive Noise	RSF tuned	RSF default	RSF noise tuned	RSF noise default	Cox	Cox Noise
0.5	0.9477	0.8571	0.8549	0.8507	0.8486	0.8555	0.8482	0.8468	0.8519	0.854	0.854
1.5	0.9471	0.8666	0.8647	0.853	0.8508	0.8811	0.8791	0.8714	0.8777	0.8552	0.8553
2.5	0.9425	0.8393	0.8374	0.8299	0.8276	0.8864	0.8873	0.8772	0.8822	0.8278	0.8287
3.5	0.9383	0.7929	0.7911	0.7881	0.7857	0.8809	0.8823	0.8722	0.874	0.7823	0.7855
4.5	0.9336	0.7457	0.7441	0.7442	0.7421	0.8732	0.875	0.8636	0.8636	0.7345	0.7417

Table S8: BS results from Simulation Scenario III

Landmark Time	True Model	MV JM	MV JM Noise	JM naiive	JM naiive Noise	RSF tuned	RSF default	RSF noise tuned	RSF noise default	Cox	Cox Noise
0.5	0.0897	0.1572	0.1588	0.162	0.1636	0.1673	0.1597	0.162	0.1593	0.1577	0.1569
1.5	0.0908	0.1505	0.152	0.1595	0.1611	0.1568	0.1421	0.1476	0.147	0.1562	0.1555
2.5	0.0953	0.1666	0.1682	0.1715	0.1733	0.1517	0.1376	0.1438	0.1472	0.171	0.1701
3.5	0.0989	0.1934	0.195	0.1938	0.1957	0.1543	0.1418	0.1483	0.1554	0.1941	0.1927
4.5	0.1028	0.2191	0.2209	0.2157	0.2177	0.1562	0.1476	0.154	0.1644	0.214	0.213

Table S9: RMSE results from Simulation Scenario III

Landmark Time	MV JM	MV JM Noise	JM naïve	JM naïve Noise	RSF tuned	RSF default	RSF noise tuned	RSF noise default	Cox	Cox Noise	
0.5	0.258	0.2609	0.2674	0.2703	0.2769	0.2625	0.2668	0.262	0.2589	0.2632	0.1569
1.5	0.2429	0.2459	0.2601	0.2634	0.255	0.2252	0.2369	0.2356	0.2537	0.2586	0.1555
2.5	0.2675	0.2704	0.2762	0.2795	0.2369	0.2057	0.2209	0.2279	0.2752	0.2814	0.1701
3.5	0.3058	0.3086	0.3068	0.3098	0.2313	0.2042	0.2197	0.2354	0.3074	0.3164	0.1927
4.5	0.3407	0.3433	0.3357	0.3384	0.2292	0.2118	0.2258	0.2479	0.3337	0.3477	0.213

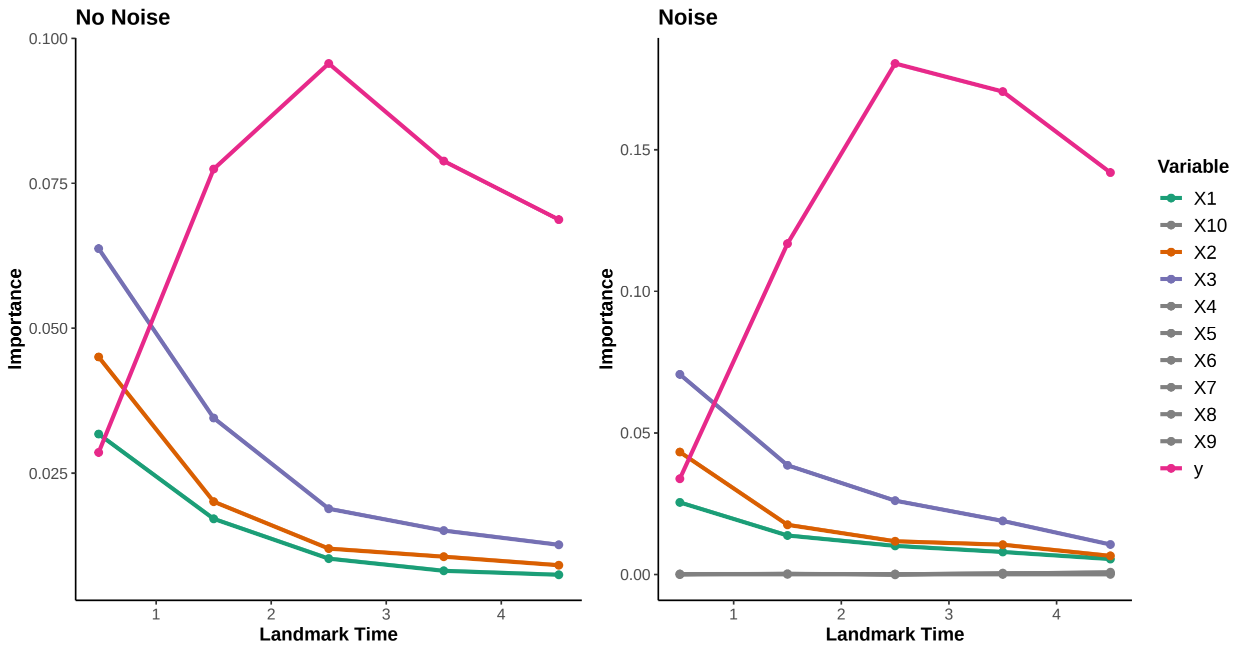

Figure S1: Simulation estimates for VIMP from Scenario II assessed at landmark times comparing importance of main effects when noise variables are included. $X1 - X3$ are baseline variables included in data generation, $X4 - X10$ are noise variables, and Y is the longitudinal variable

4 Additional Result Figures and Tables: Heart Valve Application

Table S10: Description of models fit to the heart valve data set. All models include the same base set of variables. Joint model includes the longitudinal LVMI marker. RSF and Cox models include longitudinal LVMI and EF markers, with imputation from last-observation carried forward (LOCF) or from a linear mixed effects model (LMM) with a random intercept and slope.

Model	Description	Imputation
JM	Joint model	-
Cox LMM	Cox landmark model	LMM
Cox LOCF	Cox landmark model	LOCF
RSF LMM	RSF with default parameters	LMM
RSF LOCF	RSF with tuned parameters	LOCF
Tuned RSF LMM	RSE with tuned parameters	LMM
Tuned RSF LOCF	RSE with tuned parameters	LOCF
Full RSF LMM	RSF with default parameters and all baseline covariates	LMM
Full RSF LOCF	RSF with default parameters and all baseline covariates	LOCF
Full Tuned RSF LMM	RSF with default parameters and all baseline covariates	LMM
Full Tuned RSF LOCF	RSF with default parameters and all baseline covariates	LOCF
RSF No Admin LOCF	RSF with default parameters and no administrative censoring	LOCF
RSF Tuned No Admin LOCF	RSF with tuned parameters and no administrative censoring	LOCF

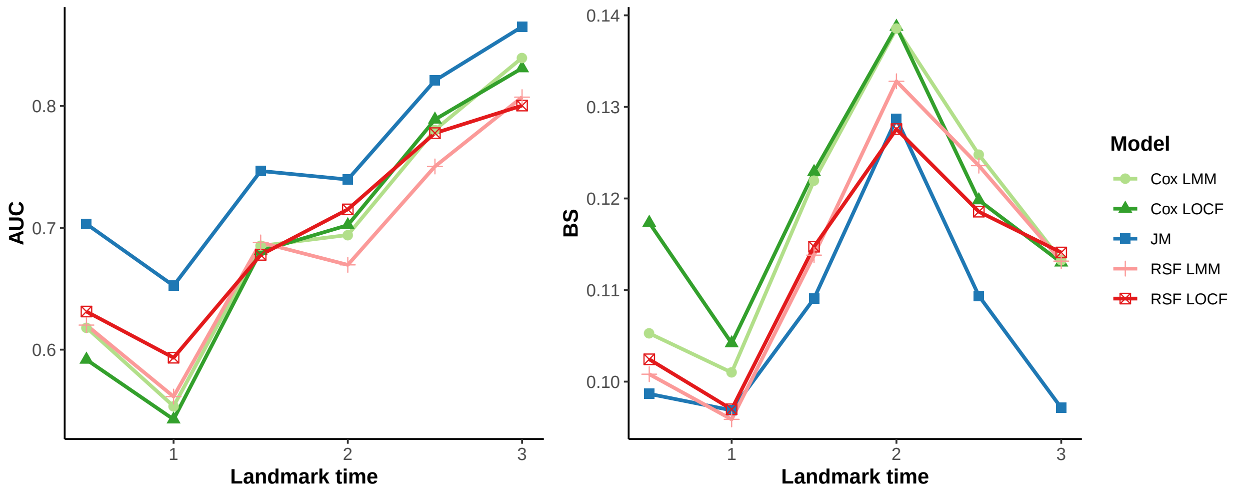

Figure S2: Heart valve application comparison of RSF Landmarking (RSF LMM, RSF LOCF) with joint model (JM) and Cox landmarking (Cox LMM, Cox LOCF). AUC shown in left panel, and BS in right panel.

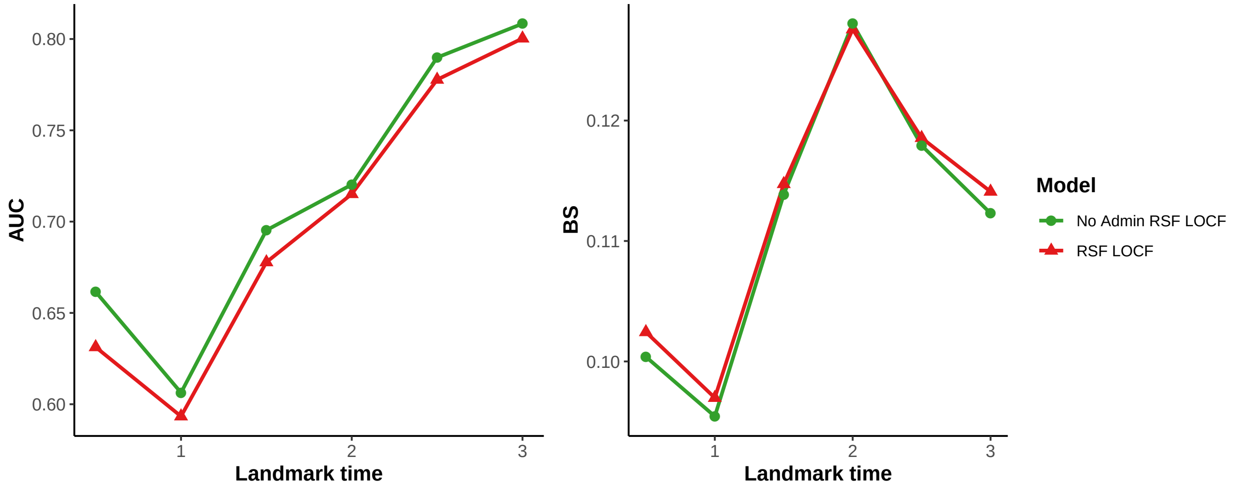

Figure S3: Heart valve application comparison of RSF models using last-observation-carried-forward imputation with (RSF LOCF) and without administrative censoring (No Admin RSF LOCF). AUC shown in left panel, and Brier score (BS) in right panel.

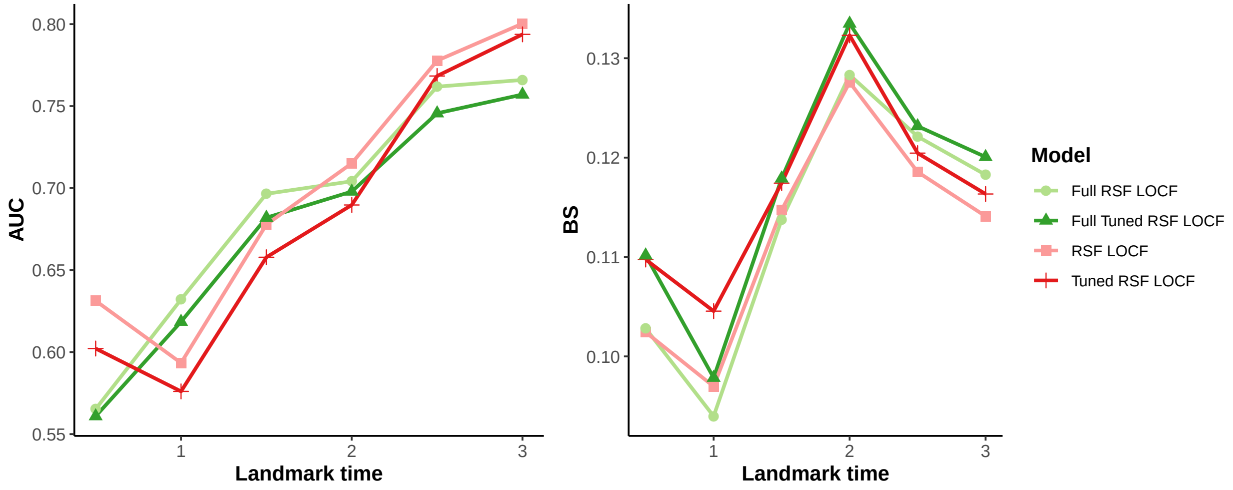

Figure S4: Heart valve application comparison of RSF models with the clinically relevant subset of covariates ("RSF") to the full RSF with all available variables ("Full RSF"). These results are presented both with tuning of parameters ("Tuned") and without.
